# Novel mitochondrial alanyl-tRNA synthetase 2 (*AARS2*) heterozygous mutations in a Chinese patient with adult-onset leukoencephalopathy

**DOI:** 10.1186/s12883-022-02720-3

**Published:** 2022-06-08

**Authors:** Yan Fan, Jinming Han, Yanyan Yang, Tuanzhi Chen

**Affiliations:** 1 Department of Neurology, Liaocheng People’s Hospital, Liaocheng, China; 2 Department of Neurology, Xuanwu Hospital, Capital Medical University, Beijing, China

**Keywords:** *AARS2*, Leukoencephalopathy, Gene mutation, White matter, Ovarian failure

## Abstract

**Background:**

Missense mutations in the mitochondrial alanyl-tRNA synthetase 2 (*AARS2*) gene are clinically associated with infantile mitochondrial cardiomyopathy or adult-onset leukoencephalopathy with early ovarian failure. To date, approximately 40 cases have been reported related to *AARS2* mutations, while its genetic and phenotypic spectrum remains to be defined.

**Case presentation:**

We identified a 24-year-old Chinese female patient with adult-onset leukoencephalopathy carrying novel compound heterozygous pathogenic mutations in the *AARS2* gene (c.718C > T and c.1040 + 1G > A) using a whole-exome sequencing approach.

**Conclusions:**

Our findings further extend the mutational spectrum of *AARS2*-related leukoencephalopathy and highlight the importance of the whole-exome sequencing in precisely diagnosing adult-onset leukoencephalopathies.

## Background

Since mitochondrial DNA (mtDNA) spans only about 16.5 kilobase pairs (kbp), it primarily relies on the nuclear-encoded proteins for transcriptional and translational regulation of its own housekeeping genes. Mutations in these mitochondrial genes are increasingly viewed as a cause of neurological disorders in humans. The alanyl-tRNA synthetase 2 (*AARS2*, OMIM612035) gene, located on chromosome 6p21.1, is a nuclear gene that encodes mitochondria-specific alanyl-tRNA synthetase enzyme, which plays a crucial role in mitochondrial translation [1]. *AARS2* gene mutations cause two separate and extremely rare clinical phenotypes, namely infantile mitochondrial cardiomyopathy [2] and adult-onset leukoencephalopathy with early ovarian failure [3, 4]. Due to the broader application of next-generation sequencing in clinical practice, we have been able to recognize the expanding phenotypic spectrum of *AARS2* mutations, including a benign phenotype without leukoencephalopathy and lethal primary pulmonary hypoplasia [5, 6]. Notably, some reported cases of familial pulmonary hypoplasia with *AARS2* mutations had been linked to the European founder (c.1774C > T variant) [5]. Phenotypic heterogeneity is also evident in individuals with identical *AARS2* variants [5]. Therefore, a better understanding of this devastating disease is urgently warranted. Here, we reported novel *AARS2* mutations [c.718C > T (p. Leu240Phe) and c.1040 + 1G > A] associated with adult-onset leukoencephalopathy and early ovarian failure in a 24-year-old female patient. Clinical features, brain magnetic resonance imaging (MRI) characteristics and genetic testing performed during the diagnosis are presented in this case study.

## Case presentation

**Fig. 1:**
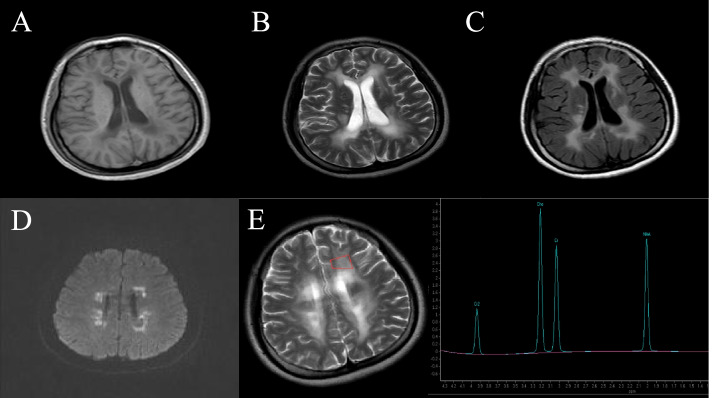
Brain magnetic resonance imaging of this patient (**A**-**D**) Axial T1W1, T2WI, fluid-attenuated inversion recovery (FLAIR) and diffuse weighted imaging (DWI), respectively. T1-hypointense and hyperintense signal abnormalities on T2W1 and FLAIR can be noted around the corpus callosum and bilateral ventricles. Diffusion restriction in selective white matter was noted. **E** Magnetic resonance spectroscopy (MRS) showed an increased Cho/Cr ratio in frontal lesions

**Fig. 2:**
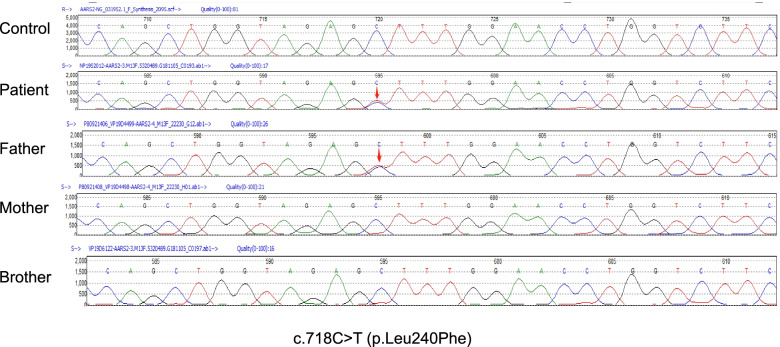
*AARS2* gene mutations (missense mutation) in the patient and her father. The patient had a missense mutation in the *AARS2* gene c.718C > T (p.Leu240Phe). The variant was transmitted paternally (red arrows)

**Fig. 3:**
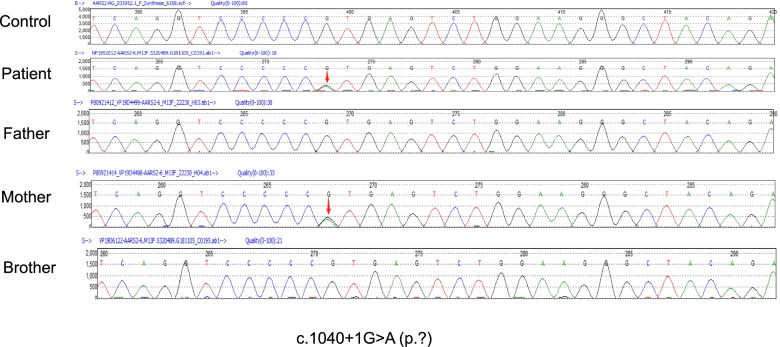
*AARS2* gene mutation (splicing mutation) in the patient and her mother. The patient had a splicing mutation in the *AARS2* gene c.1040 + 1G > A (p.?). The variant was transmitted maternally (red arrows)

A 24-year-old female patient was admitted to the Department of Neurology at Liaocheng People's Hospital due to persistent amenorrhea for 2 years and weakness of both lower limbs for the past 10 months. She had no remarkable medical history, except for raising the head and walking later than other children of the same age. There was no problem with the pregnancy or delivery and also no history of drug abuse or hallucinations. No consanguineous marriage in her family was reported. She had irregular menstruation with prolonged menstrual cycles. The first symptom of amenorrhea or absence of menstruation was recorded when the patient was 22 years old. The patient felt like stepping on cotton when walking, and it was a little bit difficult for her to stand up after sitting. At the initial phase, these clinical symptoms were slightly improved after a short period of symptomatic manifestations. However, she started to seclude herself from social activities, while her bilateral lower extremity weakness progressively deteriorated without a remitting period. She also exhibited signs of cognitive decline and abnormal mental behaviors. For example, she had a slow reaction time and always presented delayed responses to doctor’s questions. Reduced communicative participation was noted in this patient during the initial screening. Her calculating and reading abilities were also decreased progressively. She had been suffering from motor function deficits (lower limb tremor, bradykinesia and rigid muscles), requiring active physical assistance for daily activities. Upon neurological evaluation, her muscle strengths of all four limbs were graded as 4-/5. A decreased sense of vibration and numbness of lower limbs were recorded in lower extremities. Moderate station ataxia (Romberg test) and tandem walking tests were not feasible in this patient. The tendon reflexes of lower limbs were sightly active and other remarkable signs, including bilateral Babinski sign and Kernig sign, were not evident. Routine laboratory investigations indicated normal renal and hepatic functions. The endocrinological hormone profiling in the serum showed that the levels of estradiol were 33.473 pg/ml (Reference ranges, follicular phase: 26.6–161 pg/ml; luteal phase: 187–382 pg/ml; ovulation: 187–382 pg/ml; menopause: 5.37–38.4 pg/ml), luteinizing hormone was 55.7mIU/ml (Reference ranges, follicular phase: 2.58–12.1 mIU/ml; luteal phase: 0.83–15.5 mIU/ml; menopause: 13.1–86.5 mIU/ml), and follicle-stimulating hormone was 87.9 mIU/ml (Reference ranges, follicular phase: 1.98–11.6 mIU/ml; luteal phase: 1.38–9.58 mIU/ml; menopause: 21.5–131 mIU/ml). Blood tests for the levels of very-long-chain fatty acids and lysosomal enzyme activities were unremarkable. T2-weighted sagittal and fluid-attenuated inversion recovery (FLAIR) images revealed white matter hyperintensities and abnormal signals on bilateral periventricular regions, corona radiata, and corpus callosum (Fig. 1A-C). White matter rarefaction was visible on FLAIR. Restricted diffusion in selective white matter was noted (Fig. 1D). Magnetic resonance spectroscopy (MRS) showed an increased ratio of Cho and Cr in frontal lesions (Fig. 1E). Whole-exome sequencing results indicated that the exon4 of the *AARS2* gene contained a missense mutation at c.718C > T (p.Leu240Phe) in this proband (Fig. 2), when matched with the HG19 reference genome sequence and the transcript variant NM_020745.3. In silico analyses using multiple bioinformatics methods suggested this mutation as ‘probably damaging’. Furthermore, an alternative splicing mutation (c.1040 + 1G > A) was also noted (Fig. 3), as confirmed by in silico analysis (a pathological variant). Both transcript variants were not previously reported in the general population database and might have deleterious effects. The father of this patient carried a heterozygous *AARS2* gene mutation (c.1040 + 1G > A), and the mother also had a heterozygous mutation c.718C > T in the same gene. The patient’s brother did not show either of these variants. Coenzyme complex treatment and glucocorticoid therapy had no therapeutic impact on this patient. During the recent follow-up visit, her health condition was found to be worsened further, and the patient’s movement was mostly restricted to bed.

## Discussion and conclusions

In this study, we reported the case of a Chinese female patient diagnosed with adult-onset leukoencephalopathy who mainly suffered from prematurely terminated menstruation and progressive motor decline following the disease onset. Brain MRI symmetrical features suggested genetic leukoencephalopathies. Genetic analysis revealed that the patient carried novel compound heterozygous pathogenic mutations in the *AARS2* gene (c.718C > T and c.1040 + 1G > A). These compound heterozygous mutations were co-segregated with the disease phenotype in her family. Both variants were predicted to be pathogenic by in silico analysis. The c.1040 + 1G > A in the *AARS2* gene was reported to be associated with frontotemporal dementia in a Korean patient [7], while the c.718C > T has not been reported in the literature. We have carefully ruled out other possibilities based on the patient’s medical background and the presence of pathogenic mutations in the *AARS2* gene in her parents, and a definitive diagnosis of *AARS2*-related leukoencephalopathy was made.

AARS2 is a nuclear-encoded mitochondrial enzyme that is essential for the mitochondrial translation machinery. AARS2 protein contains several functional domains, such as the aminoacylation domain, editing domain, and C-terminal domain [8]. Diseases associated with the *AARS2* gene mutations include missense, nonsense and/or frameshift alterations in both alleles, consistent with an autosomal recessive inheritance, which can lead to mitochondrial respiratory chain complex deficiency and metabolic dysfunctions [9]. Pathogenic *AARS2* variants were first identified in infants suffering from cardiomyopathy since the heart has a higher content of mitochondria to meet energy demand. Patients with infantile mitochondrial cardiomyopathy caused by *AARS2* mutations usually have combined complex I and III deficiencies, while individuals with *AARS2*-related leukoencephalopathy exhibit an isolated complex IV deficiency [10–12]. Studies have suggested that infantile mitochondrial cardiomyopathy with *AARS2* gene mutations are most frequently noted in the editing domain of the enzyme that severely compromises its aminoacylation capacity [13]. By contrast, milder variants may cause a partial decrease in the AARS2’s synthetase function associated with leukoencephalopathy and premature ovarian insufficiency [5, 13].

It is important to note that patients with *AARS2*-related leukoencephalopathy may be erroneously named as hereditary diffuse leukoencephalopathy with spheroids (HDLS) due to the clinical as well as histopathological similarities between these two diseases [14, 15]. HDLS was first named by a Swedish research group in 1984 [16]. Later, the *AARS* gene mutation (p.Cys152Phe) was identified as a possible cause in the original Swedish HDLS family, who were negative for *CSF1R* gene mutation [17]. Screening for pathogenic *AARS2* gene mutations is thus needed to be recommended in selective *CSF1R*-negative leukoencephalopathy individuals [3]. Furthermore, the average age of onset of these two rare diseases is distinct, with *AARS2*-related leukoencephalopathy being 27.3 years [18] and *CSF1R*-related leukoencephalopathy being 41.4 years [19]. Our reported *AARS2*-related leukoencephalopathy patient suffered from typical clinical symptoms including ceased menstruation, cognitive decline, and decreased strength of limbs in her 20’s, which were consistent with previous case reports. However, it is important to note that the onset age has a wide range and may not be sufficient to distinguish them clinically.

*AARS2*-related leukoencephalopathy is reportedly less common than *CSF1R*-related leukoencephalopathy. With approximately 40 cases caused by *AARS2* gene mutations have been reported in the literature [20], we have just begun to recognize the wide genetic and phenotypic spectrum related to *AARS2* gene mutations. The whole-exome sequencing has emerged as a powerful approach in the diagnosis of adult-onset leukoencephalopathies, and more collaborative efforts are needed in order to improve better therapeutic management.

## Data Availability

The datasets generated during and/or analyzed during the current study are available from the corresponding author on reasonable request.

## Abbreviations

MtDNA: Mitochondrial DNA
AARS2: Alanyl-tRNA synthetase 2
MRI: Magnetic resonance imaging
FLAIR: Fluid-attenuated inversion recovery
MRS: Magnetic resonance spectroscopy
HDLS: Hereditary diffuse leukoencephalopathy with spheroids
CSF1R: Colony stimulating factor 1 receptor
